# Translational Relevance of Mouse Models of Atopic Dermatitis

**DOI:** 10.3390/jcm10040613

**Published:** 2021-02-06

**Authors:** Justin Choi, Nishadh Sutaria, Youkyung Sophie Roh, Zachary Bordeaux, Martin P. Alphonse, Shawn G. Kwatra, Madan M. Kwatra

**Affiliations:** 1 Department of Dermatology, Johns Hopkins University School of Medicine, Baltimore, MD 21231, USA; justin.choi@duke.edu (J.C.); nsutari1@jhmi.edu (N.S.); sophieroh@jhmi.edu (Y.S.R.); zbordeaux1@pride.hofstra.edu (Z.B.); malphon1@jhmi.edu (M.P.A.); 2 Department of Anesthesiology, Duke University School of Medicine, Durham, NC 27710, USA; madan.kwatra@duke.edu

**Keywords:** atopic dermatitis, eczema, mouse models, dermatology, immunology

## Abstract

The complexity of atopic dermatitis (AD) continues to present a challenge in the appropriate selection of a mouse model because no single murine model completely recapitulates all aspects of human AD. This has been further complicated by recent evidence of the distinct AD endotypes that are dictated by unique patterns of inflammation involving Th1, Th2, Th17, and Th22 axes. A review of currently used mouse models demonstrates that while all AD mouse models consistently exhibit Th2 inflammation, only some demonstrate concomitant Th17 and/or Th22 induction. As the current understanding of the pathogenic contributions of these unique endotypes and their potential therapeutic roles expands, ongoing efforts to maximize a given mouse model’s homology with human AD necessitates a close evaluation of its distinct immunological signature.

## 1. Introduction

Atopic dermatitis (AD) is a common, relapsing inflammatory skin condition characterized by pruritic, erythematous plaques and papules typically affecting the body’s flexural surfaces. While AD is known to emerge due to barrier dysfunction, aberrant immune activation, and genetic predisposition, a clear understanding of the pathogenesis of its varying clinical presentations remains under investigation. Current knowledge of AD’s multifaceted pathogenesis has been predicated on a diverse array of murine models that have played a pivotal role in delineating the functions of various susceptibility genes and exogenous triggers in the disease process.

However, the heterogeneity of AD disease in humans continues to present a challenge in selecting an appropriate mouse model for preclinical studies, given that no single model fully recapitulates all aspects of human AD. This has been further complicated by the recent identification of immunologically distinct human AD subtypes that occur due to differential inflammatory axis activation [1]. As the roles of these unique inflammatory patterns and their potential therapeutic implications in AD are further clarified, the selection of appropriate mouse models based on downstream immune pathways that modulate these clinically distinct subtypes is especially important in drug validation studies.

Thus, this review seeks to evaluate commonly used mouse models for AD and to highlight the immune pathways that are affected in mice. We hope to aid investigators in the selection of appropriate models that carefully balance immune factors alongside the underlying genetics, phenotype, and transcriptomic similarities to human AD in order to optimize their translational relevance in future studies.

## 2. An Overview of Mouse Models for Atopic Dermatitis

The current repository of AD murine models reflects a broad range of mechanisms used to induce eczematous dermatitis, including the use of exogenous agents, transgenic mice, and inbred mice. Several of these mechanisms, such as mucosa-associated lymphoid tissue lymphoma translocation protein 1 (MALT1) deficiency, fibroblast-specific inhibitor of nuclear factor kappa-beta subunit beta (Ikk2) deficiency, and Matt deficiency, have been only loosely correlated to human AD, and a clear understanding of their pathogenic contributions resulting in AD has yet to be fully delineated [2,3,4]. Nevertheless, the cutaneous inflammation observed in most models demonstrates significant overlap with key features found in human AD lesions, including elevated serum IgE, inflammatory infiltrate consisting of eosinophils, mast cells, and lymphocytes, increased epidermal thickness, hyperkeratosis, parakeratosis, acanthosis, and spongiosis [4,5,6].

More recently, transcriptomic analyses have measured similarities between highly differentially expressed genes in human AD and select murine models using the Meta-analysis derived atopic dermatitis transcriptome (MADAD), with data demonstrating most significant overlap with Adam17^fl/fl^Sox9^Cr*e*^ mice (34% overlap) and mice induced with IL-23 (36% overlap) [6,7,8]. This is followed by NC/Nga mice, demonstrating 18% overlap with the human AD transcriptome, and oxazolone-sensitized mice, with 17% overlap [7]. Similarly, gene set enrichment analysis conducted by Nunomura et al. (2019) on Ikk2-deficient (Ikk2^∆NES^) mice demonstrated a high degree of concordance with human AD in both upregulated (16 of 30) and downregulated (19 of 30) genes [3].

Consistent with the Th2 induction that broadly underscores all human AD endotypes, murine models invariably demonstrate Th2-biased immune response, with elevated levels of Th2-related cytokines: IL-4, IL-5, IL-13, and/or thymic stromal lymphopoietin (TSLP). Eight models (models 1–8) reported exclusively Th2 elevations, while two models (models 9–10) reported Th1 in addition to Th2 activation (Table 1). Four models (models 11–14) reported heightened Th2 and Th17 inflammation, three of which (models 12–14) also reported an increase in Th1-related cytokines (Table 2).

Ten models noted Th22-related T-cell and cytokine changes in mice: Adam17^fl/fl^Sox9^Cre^, NC/Nga, IL-23-induced, house dust mite (HDM)-induced, ovalbumin (OVA)-induced, chloromethylisothiazonilone or methylisothiazonilone (CMIT/MIT)-primed, oxazolone (OXA)-induced, flaky tail (*ft*), vitamin D3-induced, and K5-tTA-IL-22 mice (Table 3). These include all models whose transcriptomic homology with human AD have been evaluated: IL-23-induced (37%), Adam17^fl/fl^Sox9^Cre^ (34%), NG/Nga (18%), oxazolone-induced (17%), ovalbumin-induced (11%), and *ft* (4%) mice [7]. On the other hand, Th22 activity was assessed in Ikk2^∆NES^ mice and found to be unchanged in affected animals (Table 2). All models with Th22 induction also exhibited Th17 upregulation, while six (models 17–18, 20, 22–24) also reported Th1 inflammation. Thus, among the models evaluated for this study, six murine models demonstrated broad upregulation of Th1, Th2, Th17, and Th22 inflammation: Adam17^fl/fl^Sox9^Cre^, IL-23-exposed, OXA-induced, OVA-induced, *ft*, and vitamin D3-induced mice.

Three models are notable for outlining methods that may aid in selectively modulating inflammation: OVA with CMIT/MIT exposure, *ft* mice, and HDM-induced mice [28,36,37,38]. While ovalbumin commonly to induce eczema in mice, Go et al. (2020), found that mice sensitized with CMIT/MIT before OVA displayed an augmented Th17 reaction than mice exposed to OVA alone [36]. Likewise, Fallon et al. (2009) demonstrated higher Th17 activation in BALB/c mice harboring the *(ft)* mutation compared to C57BL/6 mice [37]. Similarly, among models that demonstrate Th22 upregulation, the HDM-induced model allows for selective suppression of Th22 response with the use of BALB/c instead of C57BL/6 mice [28]. Although these methods demonstrate the potential for modeling multiple endotypes within a single genetic strain, both OVA-induced and *ft* mice share the least homology with the human AD transcriptome, at 11% and 4%, respectively [7], while the transcriptomic homology of the HDM-induced model has not been evaluated.

Existing drug validation studies that evaluated the effects of FDA-approved and investigational therapies in select models provide insight into their translational utility (Table 4). Corticosteroids tested against NC/Nga mice and OXA-challenged mice led to improvements in histopathologic features of AD, while also reducing the expression of Th2 cytokines in NC/Nga mice and Th2/Th17-related cytokines in OXA-challenged mice [42]. Calcineurin inhibitors tacrolimus and pimecrolimus have been tested widely against NC/Nga, OXA-challenged, Ikk2∆^NES^, DNFB-challenged, and HDM-induced mice [3,43,44], with models demonstrating variable response to the indicated compounds in terms of histopathologic improvements. While treatment with tacrolimus and pimecrolimus led to reductions in Th2 and Th17 activity, inflammatory cytokine suppression was not evaluated in all tested models. Conversely, 2,4-dinitrofluoro-benzene (DNFB)-challenged and HDM-induced mice showed minimal inflammatory improvement with cyclosporine treatment, with the former demonstrating partial suppression of IL-13 and TNF-α upregulation [45]. Crisaborole and Compd3, which act via PDE4 inhibition, demonstrated efficacy against calcipotriol-induced AD lesions, demonstrating reductions in TSLP expression and skin swelling [46,47]. Novel Janus kinase (JAK) inhibitors have also been studied broadly in numerous models, including NC/Nga, Ikk2∆^NES^, DNFB-challenged, HDM-induced, and human skin-grafted mice [44,45,48,49]. Mice treated with JAK inhibitors delgocitinib and tofacitnib led to broad inhibition of Th2-related cytokines, as well as improvements in clinical severity and barrier function [44,45,48,49].

## 3. Discussion

Ongoing efforts to demystify the complexity of AD have led to the identification of immunologically distinct phenotypes that vary based on permutations of Th1, Th17, and Th22 inflammation [1]. AD in Asian patients is thought to harbor heightened Th17/Th22 bias, AD in African Americans have Th2/Th22 skewing with Th17 attenuation, and European American lesions exhibit Th1/Th17 suppression [50]. Similarly, pediatric AD is notable for an unaltered Th1 profile against a background of Th17/Th22 upregulation [51], while adult AD is characterized by broad inflammatory induction of all axes [52]. Studies have also observed immune heterogeneity across adult subpopulations when stratified by age group, noting more robust Th22 upregulation in younger adults compared to older adults [52]. The potential to tailor treatments based on a patient’s unique endotype represents an exciting new frontier in clinical drug discovery as the treatment paradigm for AD shifts from systemic interventions to targeted therapies [52].

Thus, the ability to reproduce these endotypes in mice represents a critical step toward optimizing the selection of translationally relevant mouse models, particularly in an era driven by the rising importance of personalized medicine [52]. Kim et al. (2019) previously outlined several features that warrant consideration in the selection of a mouse model, including gross phenotype/histology, serum profile, transcriptomic similarities, and immunophenotype [6]. More recently, Gilhar et al. (2020) proposed a list of criteria that animal models of human AD should meet, which included the consideration of inflammatory patterns [29]. An in-depth assessment of a model’s unique immunological signature and the ability to individually modulate specific immune axes may be key for future preclinical studies seeking to assess how novel biologics blockade the inflammatory upregulation that defines specific AD endotypes, as selection based on genetics, phenotype, or gene expression profile may not adequately capture the immunologic complexity of AD. For instance, while Ikk2^∆NES^ mice are notable for their transcriptomic homology with human AD, their lack of Th22 induction potentially limits their utility for the study of Asian, pediatric, and young adult endotypes that are notable for Th22 inflammation [3]. Similarly, NC/Nga mice characterized by high transcriptomic homology with the human AD transcriptome may be of limited translational relevance for the European American endotype given its marked Th17 induction [30,31,50].

Looking to the future, newer techniques such as the bioengineered humanized skin model [17] and the transplantation of stimulated peripheral blood mononuclear cells (PBMC) [29] hold great promise in their potential ability reproduce population-specific immune signatures in mice. While these techniques are further explored and characterized, a more practical approach to improving existing models’ translational relevance can be guided by efforts to increase their versatility by exploring modifications that selectively modulate inflammatory responses, such as the use of CMIT/MIT prior to OVA sensitization to bolster Th17 response [36], or by characterizing the inflammatory response demonstrated by different mouse strains to aid in the selection of a model that most closely mimics a desired immune signature [28,37]. For instance, researchers seeking to model the European American AD endotype, noted for its Th17 attenuation, may use C57BL/6 mice instead of BALB/c mice within the *ft* model in order to abrogate Th17 response [37]. Similarly, the Asian or pediatric endotypes may be best reproduced with HDM-induced C57BL/6 mice to bolster Th22 inflammation [37].

In both cases, a concern lies in the task of capturing the complex features of AD in their entirety, which is further complicated by the lack of consensus regarding the features that define such criteria [29]. Thus, an additional measure to optimize the utility of existing models may involve validating therapies across a heterogenous panel of animal models, taking advantage of the common mediators that underlie cutaneous inflammation in different models. Defining an agent’s pattern of anti-inflammatory activity across multiple models that encompass all relevant features of AD may overcome their individual limitations and provide a broader picture of therapeutic response.

There are several additional limitations to selecting a model based on its endotype. Many models have not been completely evaluated for their immunologic signatures, while existing drug validation studies provide only limited insight into translational utility of tested models given their incomplete profiling of inflammatory suppression. Further studies are warranted to detail the inflammatory changes in all AD mouse models, and to characterize the degree of similarity in therapeutic response between murine AD and human AD. Moreover, generalizability is bound by interspecies differences in immune systems and their relationship to relevant biological pathways [6,53]. For instance, IL-17 is mostly produced by Th17 cells in humans and by γδ T cells in mice. While the contribution of IL-17-producing γδ T cells has been assessed in human psoriasis, their role in human AD remains uncharacterized [53,54]. Similarly, although humanized skin and PBMC models represent promising strategies to recreate specific inflammatory signatures in mice, xenografts are still subject to interference caused by interactions with host physiology [17].

In conclusion, as our understanding of the specific roles of the inflammatory axes crystallizes, future studies may shift toward optimizing the efficacy of targeted therapies based on their specific effects on the immune factors that determine specific AD phenotypes. Thus, in an effort to overcome the challenge of selecting a mouse model that broadly captures the intricacy of human AD, immunophenotypic considerations should play a more central role in the selection of AD mouse models in future preclinical studies.

## Figures and Tables

**Table 1:** Mouse models with Th2 and/or Th1 upregulation.

Model	Mechanism	Features	Immune Profile	Comparison to Human AD (If Applicable)	References
(1) Stat6VT transgenic Mice	Transcriptionally active STAT6 downregulates loricrin and involucrin expression in the epidermis.	Hyperkeratosis, epidermal and dermal thickening, lymphocytic and eosinophilic infiltrate.	Th2 (IL-4)	Certain STAT6 intron SNPs with increased promoter activity were found to be associated with an increased risk of childhood AD.	[9,10,11]
(2) K5-tTA-IL-13 mice	Transgenic mice with expression of IL-13 in the skin induced with the absence of a tetracycline	Dermal and epidermal thickening, spongiosis, hyperkeratosis, mononuclear and eosinophilic infiltration	Th2 (IL-13, TSLP)		[12]
(3) K5-TSLP	Tetracycline-inducible, skin-specific transgene expressing TSLP	Acanthosis, spongiosis, hyperkeratosis, dermal mononuclear infiltrate	Th2 (IL-4, IL-5)		[13]
(4) hK14mIL33tg	Transgenic mice with skin-specific expression of IL-33 driven by the human keratin 14 promoter.	Dermatitis with infiltrations of mast cells and eosinophils; increased IgE.	Th2 (IL-5, IL-13)		[14]
(5) Card11^unm^ mice	Mice harbor Card11 single nucleotide variant that attenuates TCR/CD28 signaling to reduce the efficiency of NFκB signaling, resulting in the gradual expansion of Th2 cells.	Acanthosis, parakeratosis, and hyperkeratotic scale, and heavy infiltration by mast cells.	Th2	Dermatitis-like symptoms reported in humans with Card11 deficiency (Demeyer et al., 2019)	[15]
(6) Unmodulated mice with a mutation in Carma1/Card11	Mice with a mutation in Carma1/Card11 have impaired NFκB and JNK activation.	High serum IgE, hyperkeratosis, predominantly mast cell infiltrate.	Th2	Dermatitis-like symptoms reported in humans with Card11 deficiency (Demeyer et al., 2019)	[16]
(7) Bioengineered mouse model	Bioengineered human skin equivalents grafted onto immunodeficient mice; intradermal injection of Th2 lymphocytes induce AD phenotype.	Epidermal thickening, dermal angiogenic response.	Th2 (TSLP)	Allows for selective introduction of specific cytokines and lymphocyte subsets to replicate specific inflammatory patterns. Potential interference with mouse cytokines and immune cell populations	[17,18]
(8) Diet-induced A	Dietary deficiency of unsaturated fatty acids may impair the skin barrier function	Epidermal thickening, mast cell and eosinophilic infiltrate, increased IgE	Th2 (IL-5, IL-13)		[19]
(9) EF1α transgenic mice	Transgenic mice are driven by the promoter Eμ-Lck overexpressing IL-31	Increased pruritus, hyperkeratosis, acanthosis, mast cell proliferation, and inflammatory infiltrate	Th1 and Th2		[20]
(10) _Jak^spade/spade^	A missense mutation in JAK1, resulting in hyperactivation.	Epidermal hyperplasia, mast cell/eosinophilic/lymphocytic infiltrate.	Th2 (IL-4, IL-5, IL-13) earlyTh1/Th2 (IFN-γ) late.		[21]

AD: atopic dermatitis. TSLP: thymic stromal lymphopoietin. STAT6: signal transducer and activator of transcription 6. SNP: single nucleotide polymorphism. NFκB: nuclear factor kappa B. JNK: c-Jun N-terminal kinase.

**Table 2:** Mouse models with Th17, Th2, and/or Th1 upregulation.

Model	Mechanism	Features	Immune Profile	Comparison to Human AD (If Applicable)	References
(11) Ikk2^∆NES^	Conditional Ikk2-deficient mice that do not express Ikk2 in the dermis fibroblasts of the face; develop AD spontaneously.	Keratinocyte proliferation, mast cell/eosinophilic infiltrate, increased IgE.	Th2 (IL-4, IL-5, IL-9, IL-13, TSLP, and Postn), Th17 (IL-17a) IL-10/20 family of genes (IL-10, IL-19, IL-20, and IL-24) No change in Th1 or Th22	Unclear relevance of pathogenesis; Ikk2-deficient humans do not display AD-like phenotype; the role of fibroblasts in AD is not characterized. No barrier dysfunction; the study reports an increase in filaggrin. The transcriptomic analysis shows broad similarities with human AD.	[3]
(12) MALT-1 knockout	MALT1 KO interferes with TCR-induced gene expression, lymphocyte proliferation, and regulatory T cell development, leading to Th2 expansion.	Acanthosis, hyperkeratosis, and parakeratotic scaling, as well as CD3+ T cell infiltration.	Th1 (IFN-γ) Th2 (IL-4) Th17 (IL-17)	Dermatitis is reported in humans with MALT1 deficiency. (Demeyer et al., 2019)	[22]
(13) Tmem79/Mattrin mutants	No expression of the protein mattrin; Impaired lamellar granular secretory system, leading to dysfunctional stratum corneum.	IL-17-dependent acanthosis, orthokeratosis, inflammatory infiltrate. Higher IgE response and TEWL levels in ma/ma after challenge with house dust mite allergen compared to FLG(ft/ft) mice.	Th1 (IFN-γ) Th2 (IL-4) Th17 (IL-17A)	Matt gene mutation was found to have only a small but significant association in human AD risk.	[23,24,25,26]
(14) 2,4-dinitrofluoro-benzene	Optimized DNFB dosing/scheduling to induce AD.	Lymphocytic and mast cell infiltrate epidermal hypertrophy and edema.	Th1 (IFN-γ) Th2 (IL-4) Th17 (IL-17A)	DNFB is also used to model other proliferative skin disorders.	[27]

TEWL: trans-epidermal water loss. FLG: filaggrin. DNFB: 1-fluoro-2,4-dinitrobenzene.

**Table 3:** Th2 and/or Th1, Th17, or Th22 upregulation.

Model	Mechanism	Features	Immune Profile	Comparison to Human AD (If Applicable)	References
(15) K5-tTA-IL-22 mice	Transgenic mice with inducible expression of IL-22 in the skin	Thickening of the epidermis and dermis, spongiosis, hyperkeratosis, inflammatory cell infiltration (eosinophils, lymphocytes, macrophages, Langerhans cells, and mast cells), and dermal collagen accumulation	Th2 (IL-4, IL-13) Th17 (IL-17) Th22 (IL-22) Decreased IL-1 (low IFN-γ)		[28]
(16) NC/Nga	Spontaneous AD formation (pathogenesis undetermined)	Moderate epidermal hyperplasia with elongation of rete ridges, hyperkeratosis, increased mast cells, and eosinophils, increased IgE	Th2 (IL-4, IL-5)Th17/Th22 (IL-17A, IL-22)	18% homology with human AD transcriptome	[29,30,31]
(17) IL-23 injection in CCR2-deficient mice	IL-23 injection stimulates IL-22-dependent dermal inflammation and acanthosis; CCR2 blockade shunts immune response toward Th2 and away from Th1.	Acanthosis, hyperkeratosis, increased epidermal thickness, tissue eosinophilia.	Th1 (IFN-γ) Th2 (IL-13) Th17/Th22 (IL-17A, IL-22)	37% homology with human AD transcriptome	[32]
(18) Adam17^fl/fl^ Sox9^Cre^	Adam17 deficiency in Sox9-expressing tissue causes dysbiosis, leading to AD.	Increased TEWL, eczematous skin lesions, increased IgE, mononuclear infiltrate. Dysbiosis with increased colonization of S. aureus.	Th1 Th2 (CCL17) Th17Th22	34% homology with human AD transcriptome. Adam17 deficiency in humans leads to AD-like phenotype.	[8,33]
(19) House dust mite allergen (HDM)	Epicutaneous sensitization to HDM	Epidermal hyperplasia, spongiosis, lymphocytic infiltrate, elevated serum IgE	Th2 (IL-4, IL-5, IL-13)- BALB/c and C57BL/6 miceTh17 (IL-17) Th22 (IL-22)- C57BL/6 mice		[28,29]
(20) Ovalbumin (OVA) with mechanical barrier disruption	Tape-stripping followed by sensitization with topical or inhaled OVA.	Epidermal and dermal thickening with increased collagen deposition, infiltration of CD4+ T cells, and eosinophils, increased IgE.	Th1 (IFN-γ) Th2 (IL-4, IL-5, IL-13), Th17 (IL-17)- topical OVA Th17 (IL-17)- inhaled OVA Th22 (IL-22)	11% homology with human AD transcriptome.	[29,34,35]
(21) Chloromethyl-isothiazonilone (CMIT), methylisothia-zonilone (MIT) and Ovalbumin	CMIT/MIT with OVA leads to a more pronounced Th2 and Th17 response than OVA alone.	Increased TEWL, increased serum IgE, mast cell infiltrate.	Th2 (TSLP, IL-4, IL-6, IL-13) Th17 (IL-17A)	Ability to differentially enhance Th17 to replicate certain endotypes.	[29,36]
(22) Spontaneous recessive mouse mutant flaky tail (ft)	Expression of truncated profilaggrin with functionally absent filaggrin.	Diffuse orthokeratotic hyperkeratosis, acanthosis, infiltrating lymphocytes, eosinophils, mononuclear cells, increased TEWL.	Th1 (IFN-γ) Th2 (IL-4, IL-5, IL-13), Th17 (IL-17) upon percutaneous allergen exposure with OVA Th22 (IL-22) Differences in immune upregulation depending on mouse strain: C57BL/6: Th1. BALB/c: Th2/Th17	Filaggrin is the only functionally characterized gene in human AD. 4% homology with human AD transcriptome.	[29,37,38]
(23) Oxazolone (OXA)	Chronic exposure to OXA (vs. allergic contact dermatitis).	Dermal infiltration of Th2 lymphocytes, mast cells, eosinophils, elevated IgE, epidermal hyperplasia, decreased expression of filaggrin, loricrin, and involucrin. Decreased stratum corneum ceramide content, decreased stratum corneum hydration, transepidermal water loss, and impaired lamellar body secretion.	Th1 (IFN-γ) Th2 (IL-4, IL-13) Th17 (IL-17) Th22 (IL-22)	17% homology with human AD transcriptome.	[29,34,39,40]
(24) Vitamin D3 administration	Vitamin D3 or its analog MC903 (calcipotriol) induces overexpression of TSLP	Epidermal hyperplasia, dermal inflammatory infiltrate of eosinophils, CD3, CD4, CD11c, mast cells	Th1/Th2 mixed (TSLP, IL-4, IL-5, IL-13, IL-31, IL-10, IL-8, IFN-γ, TNF) Th17 (IL-17) Th22 (IL-22)		[29,41]

**Table 4:** The effect of select FDA-approved or investigational agents on specific models.

Model	Therapeutic Agent	Class	Effects on Mice	Reference
NC/Nga	Dexamethasone	Corticosteroid	Reduction of Th2- (IL-4, IL-5) and Th17-related (IL-17A) cytokines. Reduction in tissue swelling and immune cell infiltration.	[42]
NC/Nga	Delgocitinib (JTE-052)	JAK inhibitor	Improved clinical score, decreased TEWL, restoration of hygroscopic amino acids needed for stratum corneum hydration	[48]
NC/Nga	Tacrolimus	Calcineurin inhibitor	Reduction of Th1- (IFN-γ), Th2- (IL-5, IL-13), Th17-related (IL-17) cytokines	[44]
MC903 (calcipotriol)	Crisaborole	PDE4 inhibitor	Reduction in ear thickness and skin swelling.	[46]
MC903 (calcipotriol)	Compd3	Novel PDE4 inhibitor	Reduction in TSLP expression	[47]
Oxazolone-challenged	Pimecrolimus Methylprednisolone	Calcineurin inhibitor Corticosteroid	Decrease in TEWL and increased stratum corneum hydration Reduced expression of IL-1α, TNF-α, PAR-2, and TSLP	[43]
Ikk2^∆NES^	Tacrolimus Tofacitinib Stattic	Calcineurin inhibitor JAK inhibitor Stat3 inhibitor	Partial decrease in the infiltration of leukocytes and eosinophils; partial decrease in epidermal swelling.	[3]
DNFB-challenged	Cyclosporine	Calcineurin inhibitor	Partial suppression of IL-13 and TNF-α upregulation. No effect on inflammatory changes.	[45]
DNFB-challenged	Delgocitinib (JTE-052)	JAK inhibitor	Reduction in IL-4, IL-13, and TNF-α expression. Reduction in acanthosis, spongiosis, and inflammatory infiltrate.	[45]
House dust mite allergen	Cyclosporine	Calcineurin inhibitor	No effect on ear thickness	[45]
House dust mite allergen	Delgocitinib (JTE-052)	JAK inhibitor	Reduction in ear thickness with greater efficacy than cyclosporine.	[45]
House dust mite allergen	Tofacitinib	JAK inhibitor	Diminished IL-1β, TNF-α, TSLP, IL-4, IL-13	[49]
Human skin graft model	Delgocitinib (JTE-052)	JAK inhibitor	Increased FLG protein expression	[48]

JAK: Janus kinase.
